# RUNX3 regulates renal cell carcinoma metastasis via targeting miR-6780a-5p/E-cadherin/EMT signaling axis

**DOI:** 10.18632/oncotarget.13205

**Published:** 2017-11-08

**Authors:** Feifei Chen, Xin Liu, Qian Cheng, Shudong Zhu, Jin Bai, Junnian Zheng

**Affiliations:** ^1^ Jiangsu Cancer Biotherapy Institute, Xuzhou Medical College, Xuzhou, Jiangsu, P.R. China; ^2^ Jiangsu Center for the Collaboration and Innovation of Cancer Biotherapy, Cancer Institute, Xuzhou Medical College, Xuzhou, Jiangsu, China; ^3^ Center of Clinical Oncology, Affiliated Hospital of Xuzhou Medical College, Xuzhou, China; ^4^ State Key Laboratory of Medical Genetics, School of Life Sciences, Central South University, Changsha, China; ^5^ Department of Urology, Affiliated Hospital of Xuzhou Medical College, Xuzhou, China

**Keywords:** RUNX3, renal cell carcinoma, tumor suppressor, E-cadherin, miR-6780a-5p

## Abstract

Runt-related transcription factor 3 (RUNX3) is a tumor suppressor in many human solid tumors. In this study, renal cell carcinoma (RCC) microarray analysis showed that the level of RUNX3 expression was lower in RCC tissue than in adjacent normal renal tissues, and was correlated with depth of invasion (pT stage) (*P*<0.001) and Tumor Node Metastasis (TNM) stage (*P*<0.001). RUNX3 expression was negatively correlated with poor 5-year overall and disease-free patient survival. RUNX3 suppressed RCC metastasis and invasion and increased levels of E-cadherin, an important marker of epithelial-mesenchymal transition, *in vitro* and *in vivo*. RUNX3 also inhibited microRNA-6780a-5p, which directly targeted the E-cadherin 3’untranslated region and decreased its expression. We confirmed that miR-6780a-5p mimics abrogated RUNX3-mediated E-cadherin upregulation and RCC metastasis/invasion inhibition. Thus, RUNX3 targeted the miR-6780a-5p/E-cadherin/EMT signaling axis to suppress renal carcinoma cell migration and invasion. This pathway illustrates a new RUNX3 function and provides potential targets for the treatment of RUNX3 mutant and loss-of-function RCC tumors. RUNX3 may also act as an effective prognostic indicator in RCC.

## INTRODUCTION

Renal cell carcinoma (RCC), the most prevalent malignancies of the adult kidney, reportedly accounts for approximately 90% of renal cancer patients [1]. At diagnosis, about 30% of patients have metastasic disease. RCC is resistant to conventional therapies such as radiation, hormone treatment and chemotherapy [2], and surgery to remove tumor remains the most effective method for the treatment of localized primary RCC. Unfortunalely, a higher percentage of patients develop metachronous metastases after nephrectomy [3]. Therefore, appropriate RCC biomarkers, which are critical to disease prediction, evaluation and therapy development, are urgently needed.

Runt domain family members, RUNX1, RUNX2 and RUNX3, share an evolutionarily conserved 128 amino-acid region, named the runt domain, required for formation of a stable complex with PEBP2b/CBFb and subsequent transactivation activity [4]. All three RUNX family members regulate gene expression in cell proliferation and differentiation pathways in humans [5] through binding to gene promoters or enhancers [6]. RUNX3 regulates gastrointestinal system growth in Cnidarians, one of the most primitive animals. *RUNX3* is localized on chromosome 1p.13-p36.11, where loss of heterozygosity (LOH) is frequently observed in multiple cancers, demonstrating its potential role as a tumor suppressor [7]. The RUNX3 tumor suppressor was associated with gastric cancer development about a decade ago [8], and has since been reported as inactivated in various types of invasive and pre-invasive epithelial and mesenchymal tumors [6]. RUNX3 contributes to tumorigenesis and metastasis at different levels, such as EMT [9], adhesion [10], migration and invasion [11]. However, the mechanisms of RUNX3 tumorigenesis and metastasis promotion are as yet unclear.

EMT, during which epithelial cells differentiate into motile mesenchymal cells, is considered an integral event in normal development and wound healing and is involved in progression of cancer and fibrosis [12–14]. E-cadherin, a vital hallmark of EMT, is downregulated in multiple human cancers [15, 16]. In addition to function as gene expression regulators during EMT, micro-RNAs (miRNAs) also regulate the epithelial phenotype and EMT by selectively binding mRNAs and inhibiting their translation or promoting their degradation [17]. In this study, we investigated the relationship between RUNX3 expression and clinicopathologic features, patient survival by a renal cancer tissue microarray. RUNX3 downregulation was associated with RCC features and progression. *In vitro* and *in vivo* experiments confirmed that RUNX3 suppressed RCC metastasis and increased E-cadherin expression. We found that miR-6780a-5p promoted renal carcinoma cell metastasis at least in part by directly targeting E-cadherin. RUNX3 binds the miR-6780a-5p promoter and downregulates its expression. Moreover, we demonstrated that miR-6780a-5p overexpression inhibited RUNX3-mediated E-cadherin upregulation and suppression of RCC metastasis. Thus, RUNX3 targeted the miR-6780a-5p/E-cadherin/EMT signaling axis to suppress renal carcinoma cell metastasis. This signal aix illustrates a novel RUNX3 function and provides a potential therapeutic target for RCC.

## RESULTS

### RUNX3 is downregulated human RCC

**Figure 1:**
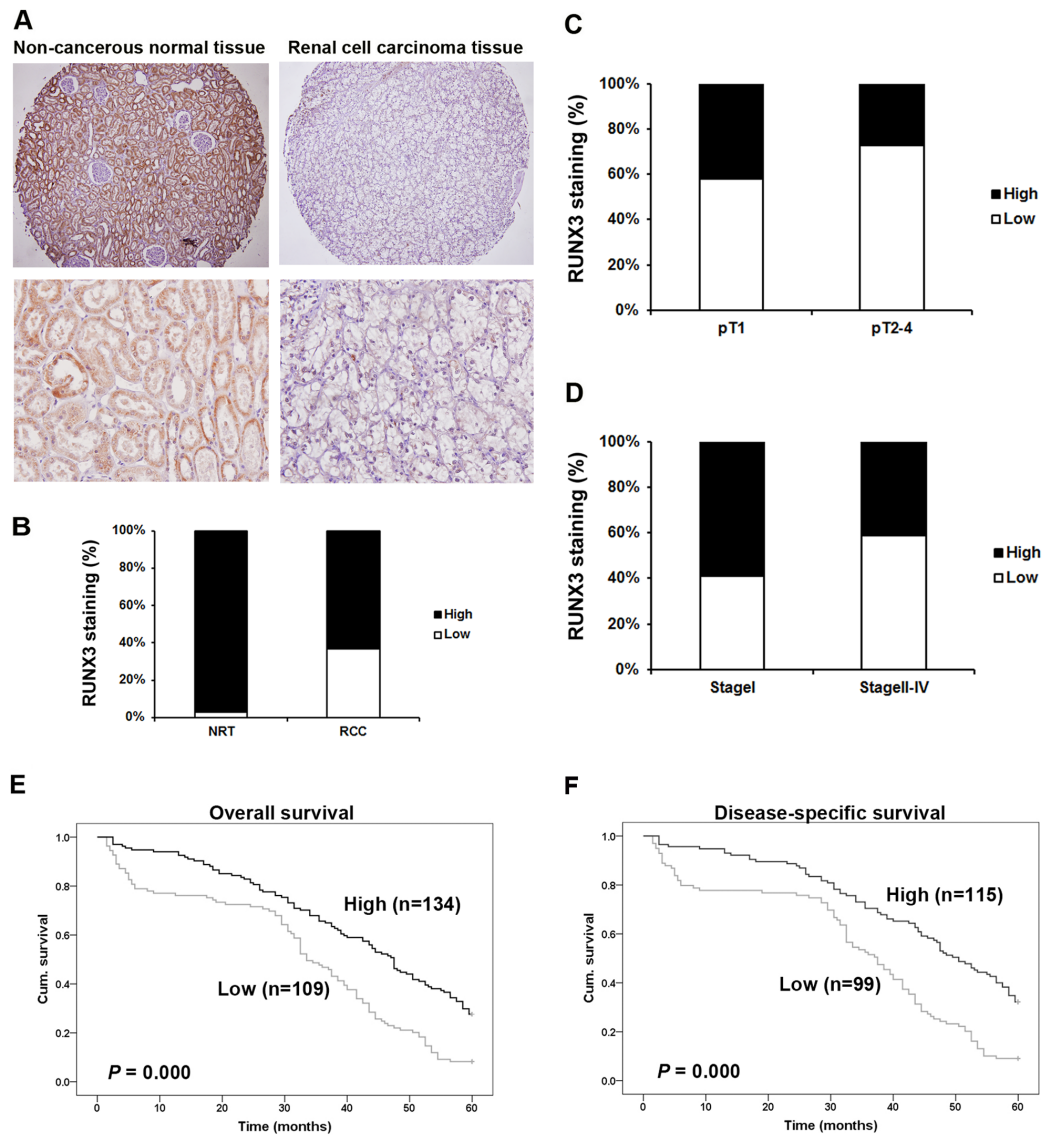
Correlation between RUNX3 expression and RCC clinicopathologic parameters Representative IHC photographs taken at different magnifications in RCC and adjacent normal renal tissues showed RUNX3 staining in TMA (Top ×100, bottom ×400) **A.** RUNX3 expression in RCC tissues was lower than in adjacent normal renal tissues (*P*<0.001, χ^2^ test) **B.** Low RUNX3 expression was correlated with depth of invasion (*P*<0.001, χ^2^ test, comparing pT1 versus pT2–pT4) **C.** and TNM stage (*P*<0.001, c^2^ test, comparing I versus II-IV) **D.** Kaplan–Meier curves of 5-year OS (*P*<0.001, log rank test) **E.** and DSS (*P* <0.001, log rank test) **F.** according to RUNX3 expression in 243 RCC patients.

Immunohistochemical (IHC) staining was employed using a tissue microarray (TMA) slide containing cancer tissues and paired normal tissues (Figure 1). RUNX3 predominantly localized to the cytoplasm and nuclei of kidney proximal tubular cells in noncancerous tissues. RUNX3 protein was reduced in tumors compared with paired normal tissues (*P* <0.001, χ^2^ test, Figure 1A–1B).

### Correlation of RUNX3 with RCC patient clinicopathologic parameters

The clinicopathologic parameters of 300 RCC patients are summarized in Table 1. RUNX3 expression was negatively related with depth of invasion (pT status; comparing pT1 versus pT2-pT4) (*P*<0.001, Figure 1C) and with TNM stages II-IV compared with stages I (*P*<0.001, Figure 1D). We did not discover significant correlations between staining of RUNX3 and patient sex, age, tumor volume, or tumor grade (Table 1).

**Table 1:** RUNX3 staining and clinicopathological characteristics of 300 renal cancer patients

Variables	RUNX3 staining			
	Low (%)	High (%)	Total	*P* ^*^
**Age**				
≤56 years	65 (45.8)	77 (54.2)	142	0.414
>56 years	64 (40.5)	94 (59.5)	158	
**Gender**				
Male	83 (41.1)	119 (58.9)	202	0.381
Female	46 (46.9)	52 (53.1)	98	
**Tumor size**				
≤7 cm	99 (42.1)	136 (57.9)	235	0.574
>7 cm	30 (46.2)	35 (53.8)	65	
**Grade**				
I	3 (18.8)	13 (71.2)	16	0.765
II	56 (40.3)	83 (59.7)	139	
III	49 (39.8)	74 (60.2)	123	
IV	10 (45.4)	12 (54.6)	22	
**pT status**				
pT_1_	78 (39.4)	120 (60.6)	198	<0.001
pT_2_	22 (46.8)	25 (53.2)	47	
pT_3_	27 (60.0)	18 (40.0)	45	
pT_4_	8 (80.0)	2 (20.0)	10	
**pN status**				
N_0_	115 (42.0)	159 (58.0)	274	0.370
N_1_-N_3_	10 (50.0)	10 (50.0)	20	
**pM status**				
M_0_	117 (46.6)	134 (53.4)	251	0.728
M_1_	3 (37.5)	5 (62.5)	8	
**TNM stage**				
I	73 (40.8)	106 (59.2)	179	<0.001
II	19 (50.0)	19 (50.0)	38	
III	24 (63.2)	14 (36.8)	38	
IV	8 (72.7)	3 (27.3)	11	

^*^Two sided Fisher's exact tests.

Some cases were not available for the information.

### RUNX3 downregulation is associated with poor patient survival

Overall survival (OS) and disease-specific survival (DSS) were employed. There were 243 and 214 overall and disease-specific mortality events, respectively. Follow-up time length was 5 years. Kaplan-Meier survival analyses revealed reduced 5-year overall (*P*<0.001, log rank test) and disease-specific (*P*<0.001, log rank test) survival times in patients with RUNX3 negative tumors compared with RUNX3 positive patients (Figure 1E–1F).

We also examined whether RUNX3 expression was an independent RCC prognostic factor. We employed univariate and multivariate Cox regression analyses with RUNX3 expression, tumor volume, age, pT status, pN status and TNM stage. Both univariate and multivariate Cox regression analyses revealed that RUNX3 expression was an independent prognostic marker for RCC patient OS (univariate: hazard ratio 0.320, 95% CI 0.165-0.621, *P*=0.001; multivariate: hazard ratio 0.315, 95% CI 0.163-0.607, *P*=0.001) and DSS (univariate: hazard ratio 0.173, 95% CI 0.078-0.383, *P*<0.001; multivariate: hazard ratio 0.182, 95% CI 0.083-0.398, *P*<0.001) (Tables 2 & 3). Our results confirmed that RUNX3 downregulation was associated with poor prognosis.

**Table 2:** Univariate Cox proportional regression analysis on 5-year overall and disease-specific survival of 300 renal cancer patients

Variable^*^	Overall survival			Disease-specific survival		
	Hazard ratio	95% CI^†^	P^*^	Hazard ratio	95% CI^†^	P^*^
RUNX3						
Low	1.000		0.001	1.000		0.000
High	0.320	0.165-0.621		0.173	0.078-0.383	
Age						
≤56 years	1.000		0.966	1.000		0.662
>56 years	0.986	0.718-1.375		0.847	0.401-1.786	
Tumor size						
≤7 cm	1.000		0.031	1.000		0.018
>7 cm	1.433	1.129-1.863		1.643	1.442-2.493	
pT status						
pT_1_-pT_2_	1.000		0.003	1.000		0.002
pT_3_- pT_4_	1.750	1.116-2.846		1.766	1.103-2.848	
pN status						
pN_0_	1.000		0.000	1.000		0.000
pN_1_- pN_3_	6.788	3.488-8.523		3.543	2.728-7.236	
TNM stage						
I-II	1.000		0.008	1.000		0.005
III-IV	1.838	1.201-3.214		1.872	1.328-3.223	

^*^P values are from Log-rank test.

^†^ CI: confidence interval.

**Table 3:** Multivariate Cox regression analysis on 5-year overall and disease-specific survival of 300 renal cancer patients

Variable^*^	Overall survival			Disease-specific survival		
	Hazard ratio	95% CI^†^	*P*	Hazard ratio	95% CI	*P*
RUNX3	0.315	0.163 to 0.607	0.001	0.182	0.083 to 0.398	0.000
Age	0.970	0.515 to 1.827	0.925	0.884	0.423 to 1.846	0.743
Tumor size	1.473	1.004 to 2.269	0.041	1.532	1.205 to 2.636	0.015
TNM stage	1.653	1.476 to 3.135	0.016	1.721	1.213 to 2.437	0.018

^*^Coding of variables: RUNX3 was coded as 1 (low), and 2 (high). Age was coded as 1 (≤56 years), and 2 (>56 years). Tumor size was coded as 1 (≤7 cm), and 2 (>7 cm). TNM stage was coded as 1 (I-II), and 2 (III-IV).

^†^ CI: confidence interval.

### RUNX3 negatively regulates RCC cell migration and invasion *in vitro*

**Figure 2:**
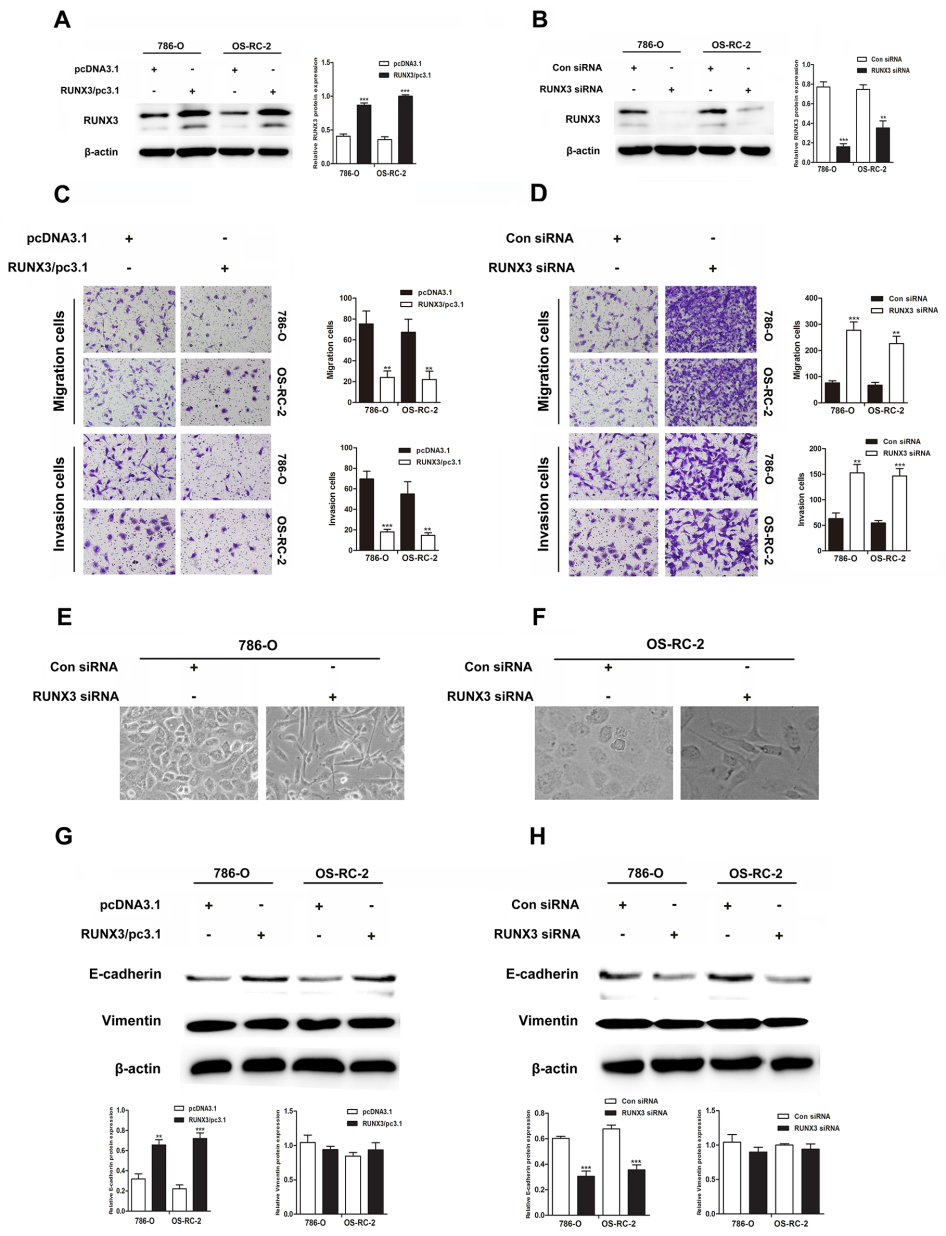
RUNX3 negatively regulates renal carcinoma cell motility and activates E-cadherin expression Western blotting showed that 24 or 48 h after transfection, RUNX3 was overexpressed or decreased as expected in renal carcinoma cells **A & B.** β-actin was used as an internal control. RUNX3 overexpression inhibited migration and invasion in 786-O and OS-RC-2 cells **C.** RUNX3 knockdown promoted migration and invasion **D.** All experiments were performed in triplicate. Data are shown as means ± SD (^**^P<0.01, ^***^P<0.001). EMT-like morphological changes in 786-O and OS-RC-2 cells transfected with control siRNA or RUNX3 siRNA **E & F.** Cellular morphology was examined under light microscopy after 48 h. Western blot analyses for E-cadherin and vimentin in 786-O and OS-RC-2 cells transfected with pcDNA3.1 or RUNX3/pc3.1 or control siRNA or RUNX3 siRNA **G & H.**

We transiently transfected 786-O and OS-RC-2 cells with control or RUNX3 plasmids, control siRNA or RUNX3 siRNA. RUNX3 was considerably up or downregulated in RCC cells 24 or 48 h after transfection (Figure 2A–2B). RUNX3 overexpression suppressed migration and invasion in both cancer cells. On the contrary, RUNX3 inhibition accelerated cell migration and invasion (Figure 2C–2D).

### RUNX3 upregulates E-cadherin expression

RUNX3 knockdown changed the morphology of RCC cells from a paving stone, sheet-like structure to a fibroblast-like spindle shape (Figure 2E–2F). Well-organized cell-cell adhesion and mesenchymal cell characteristics were absent. Because EMT activation results in tumor cell migration and invasion, our group measured levels of the E-cadherin and vimentin, which are markers for epithelial and mesenchymal, in cells transfected with control or RUNX3 plasmids or control siRNA or RUNX3 siRNA. RUNX3 positively regulated E-cadherin expression, but vimentin was not changed (Figure 2G–2H).

### RUNX3 overexpression inhibits RCC metastasis *in vivo*

**Figure 3:**
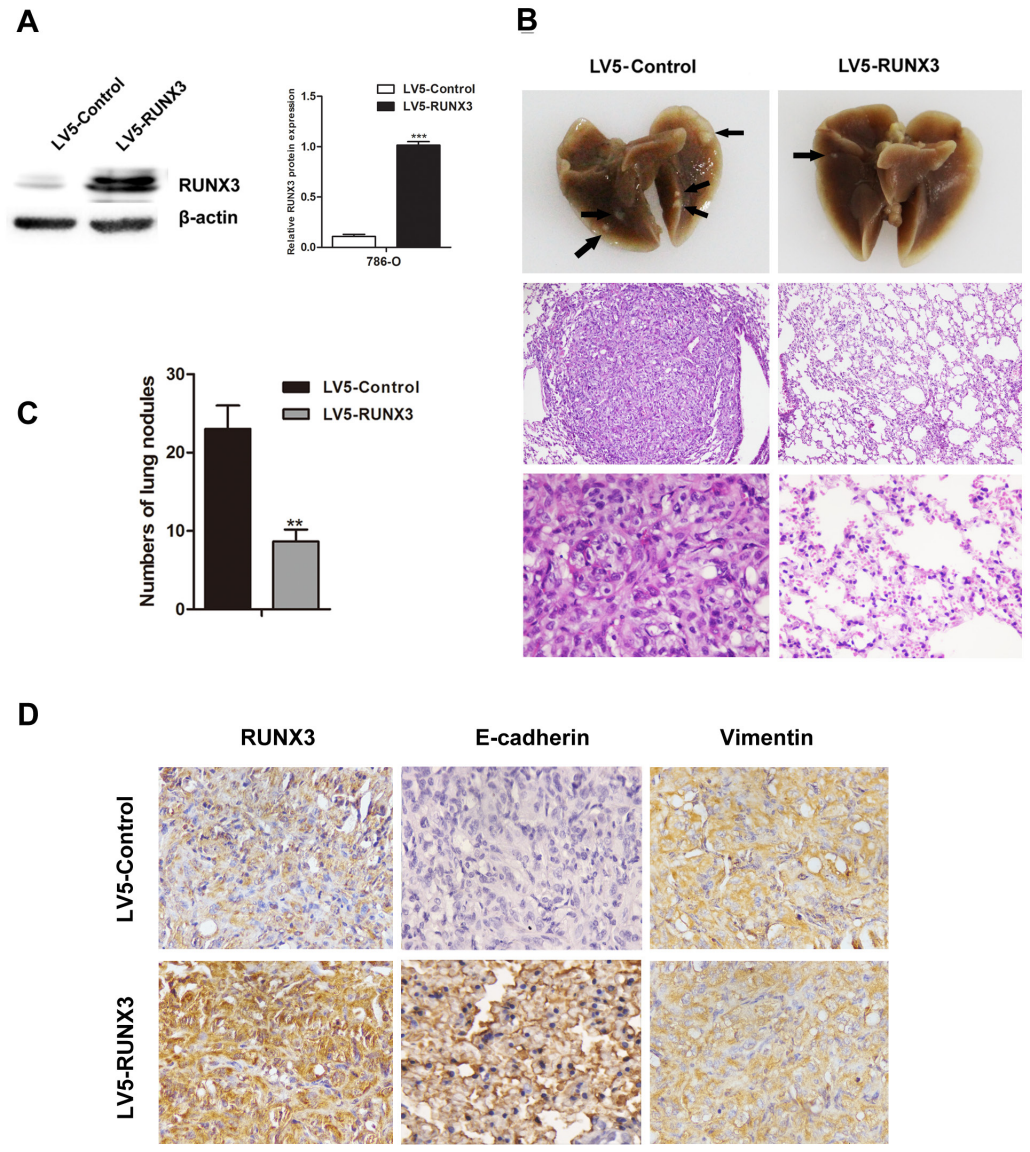
RUNX3 suppressed RCC metastasis *in vivo* Nude mice were injected intravenously with 786-O cells stably expressing LV5-RUNX3 or LV5-Control. Western blot was used to examine RUNX3 level **A.** Representative image of lung with metastatic nodules (top) and H&E staining (middle ×100, bottom ×400) 2 months after injection **B.** Arrows indicate metastatic nodules. The number of lung metastatic nodules was counted under a dissecting microscope **C.** Fewer lung metastases were seen in the LV5-RUNX3 group compared with controls. Data are shown with means ± SD (^**^P<0.01). Immunostaining of RUNX3, E-cadherin and vimentin in metastatic nodules of LV5-RUNX3 or LV5-Control groups **D.** E-cadherin expression was higher in the LV5-RUNX3 group compared with controls; vimentin was unchanged.

RUNX3 was stably overexpressed in 786-O cells (Figure 3A). Two groups of nude mice were injected with 786-O cells infected with LV5-Control or LV5-RUNX3 via tail vein. Mice were euthanized at 60 days post-implantation, and their lungs were surgically removed and fixed in 10% formal dehyde for metastatic nodule counting and histopathological analysis. We found fewer and smaller detectable tumor nodules in lungs of the LV5-RUNX3 group, and metastases were reduced as compared with the control group (Figure 3B–3C).

IHC staining of metastatic nodules in lungs showed that E-cadherin expression in the LV5-RUNX3 group was high compared with the control group, but vimentin expression was unchanged (Figure 3D).

### RUNX3 directly binds the miR-6780a-5p promoter

**Figure 4:**
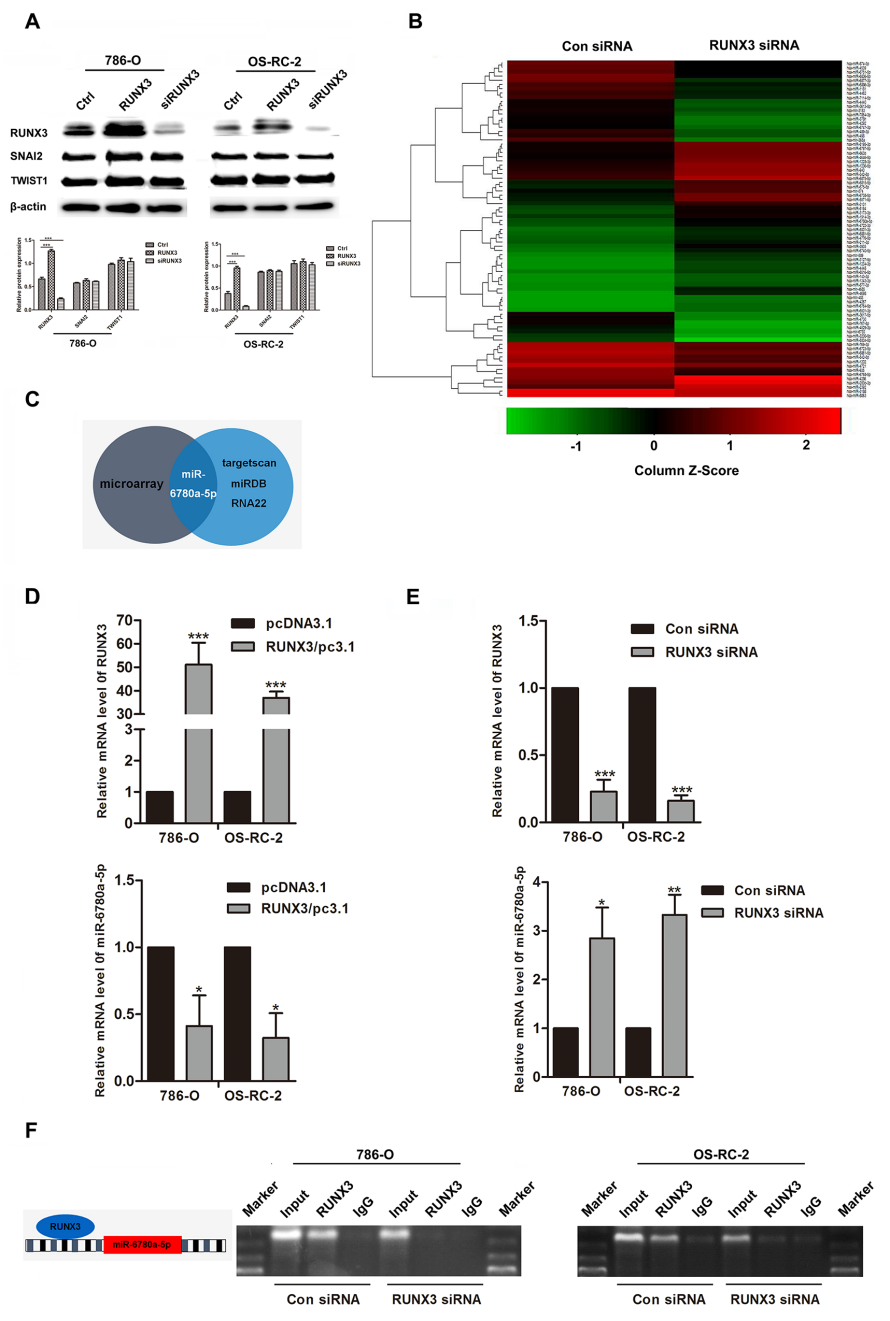
RUNX3 negatively regulated miR-6780a-5p expression in renal carcinoma cells Western blotting for RUNX3, SNAI2 and TWIST1 in 786-O and OS-RC-2 cells from the Ctrl, RUNX3/pc3.1 and siRUNX3 groups **A.** SNAI2 and TWIST1 expression were unchanged. Heat map showing differentially regulated miRNAs in 786-O cells infected with control or RUNX3 siRNA **B.** Schema for identification of putative miRNA(s) that could be activated by RUNX3 knockdown and were partly complementary to a conserved site within the E-cadherin 3’ UTR **C.** qRT-PCR analysis of RUNX3 and miR-6780a-5p levels in 786-O and OS-RC-2 cells transfected with RUNX3 overexpression plasmid or RUNX3 siRNA **D & E.** Data were means ±SD (^*^P<0.05, ^**^P<0.01, ^***^P<0.001) from three experiments. Diagram of the miR-6780a-5p promoter and RUNX3 binding sites (left) ChIP analysis in 786-O and OS-RC-2 cells 48 h after infection with control siRNA or RUNX3 siRNA **F.**

SNAI2 and TWIST1 levels, two important E-cadherin transcription factors, were not affected by RUNX3 up- or downregulation (Figure 4A). Because miRNAs have been shown to play roles in EMT and mesenchymal-epithelial transition (MET), we used a miRNA array to analyze the miRNA profiles of 786-O RUNX3 knockdown and control cells. We found that 43 miRNAs were upregulated more than two fold in 786-O RUNX3 knockdown cells (Figure 4B). We used the computational algorithms RNA22, miRDB and targetscan to analyze differentially expressed miRNAs. Only miR-6780a-5p was partially matched to the conserved site in the E-cadherin 3’-UTR (Figure 4C). We used qRT-PCR to detect miR-6780a-5p levels in RCC cancer cells 786-O and OS-RC-2 with RUNX3 overexpressed or knocked down. RUNX3 upregulated miR-6780a-5p expression while RUNX3 knockdown downregulated miR-6780a-5p (Figure 4D–4E).

We analyzed the upstream region of miR-6780a-5p and discovered one RUNX3 consensus binding sequences (Figure 4F). Chromatin immunoprecipitation (ChIP) was employed and the data displayed that RUNX3 could bind the miR-6780a-5p promoter region. When inhibition of RUNX3 expression in RCC cancer cells, immunoprecipitated DNA from the miR-6780a-5p promoter was reduced (Figure 4F).

### miR-6780a-5p downregulates E-cadherin by binding the E-cadherin 3’ UTR

**Figure 5:**
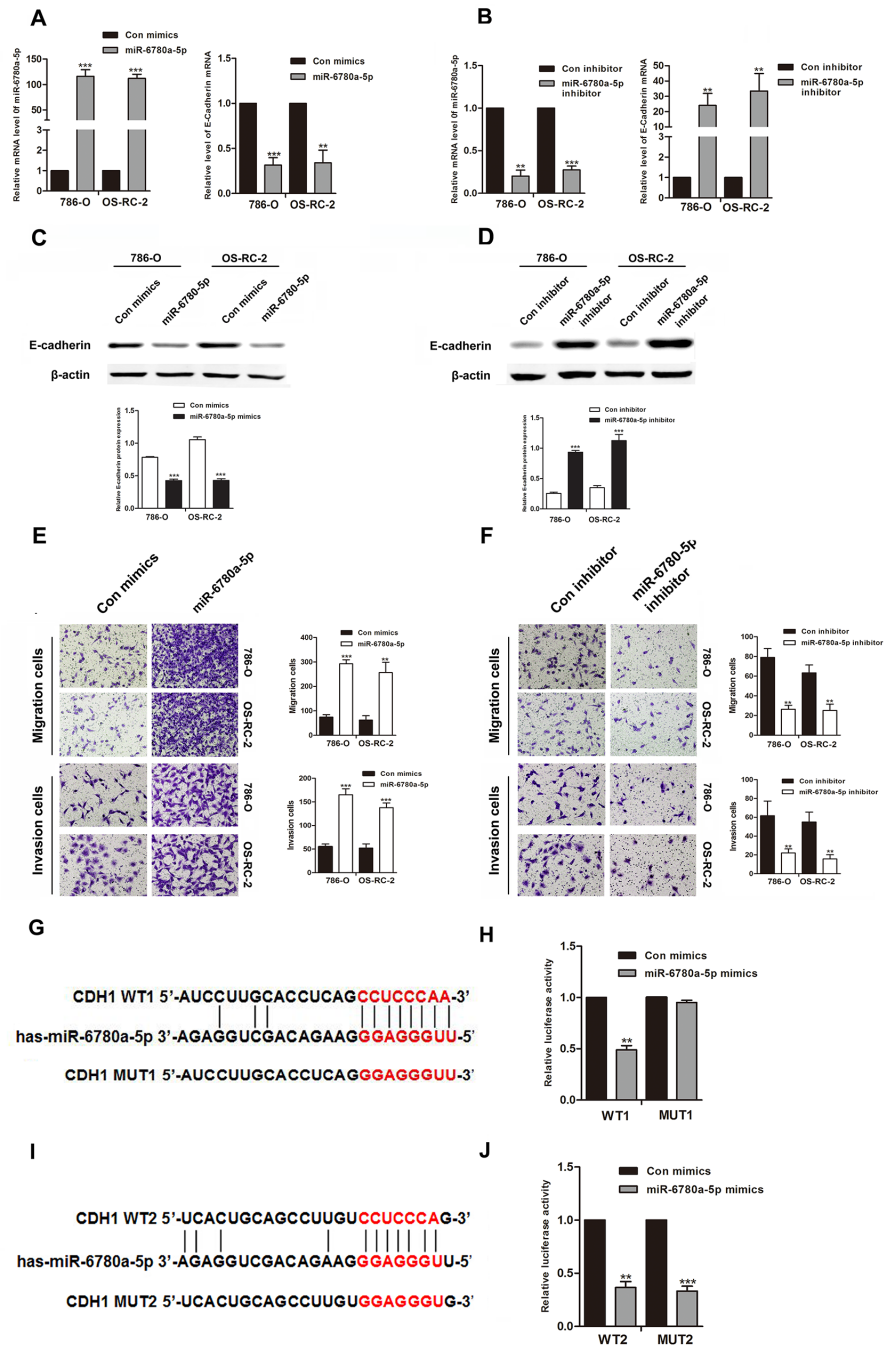
MiR-6780a-5p downregulated E-cadherin expression by directly targeting its 3’-UTR qRT-PCR and western blotting for E-cadherin in 786-O and OS-RC-2 cells transfected with miR-6780a-5p mimics or inhibitor **A-D.** MiR-6780a-5p overexpression promoted migration and invasion in 786-O and OS-RC-2 cells **E.** miR-6780a-5p knockdown inhibited migration and invasion **F.** The two predicted sites of miR-6780a-5p binding to the E-cadherin 3’-UTR were detected using bioinformatics tools **G & I.** Mutated sites in the E-cadherin 3’-UTR are shown. The effect of miR-6780a-5p on luciferase activity induced by the psiCHECK™-2-CDH1-WT1, psiCHECK™-2-CDH1-mMUT1, psiCHECK™-2-CDH1-WT2 and psiCHECK™-2-CDH1-MUT2 reporter plasmids in 786-O cells **H & J.** Data are shown as means ± S.D. of three replicates (^**^P<0.01, ^***^P<0.001).

We employed western blot to determine the influence of miR-6780a-5p on endogenous expression of E-cadherin protein. Transfection of miR-6780a-5p mimics into 786-O or OS-RC-2 cells decreased E-cadherin expression and promoted RCC cells metastasis. Conversely, miR-6780a-5p inhibitor increased E-cadherin expression (Figure 5A–5D) and suppressed RCC cells migration and invasion (Figure 5E–5F). In order to verify whether miR-6780a-5p targets CDH1 mRNA, the 3’-UTR sequence of E-cadherin was cloned into the psiCHECK™-2 vector. Forced expression of miR-6780a-5p suppressed luciferase activity of this reporter, displaying that miR-6780a-5p inhibits 3’-UTR function of CDH1. To further determine whether miR-6780a-5p specifically suppressed CDH1 by its potential binding site of seed sequence, the mutated (mutant type, MUT) reporter at miR-6780a-5p binding site was constructed. However, our data showed that activity of the MUT1 3’-UTR reporter was unchanged (Figure 5G–5I). These results illustrated that miR-6780a-5p could directly target the 3’-UTR of E-cadherin and downregulated E-cadherin expression.

### miR-6780a-5p restoration attenuates RUNX3-mediated E-cadherin expression and metastasis suppression

**Figure 6:**
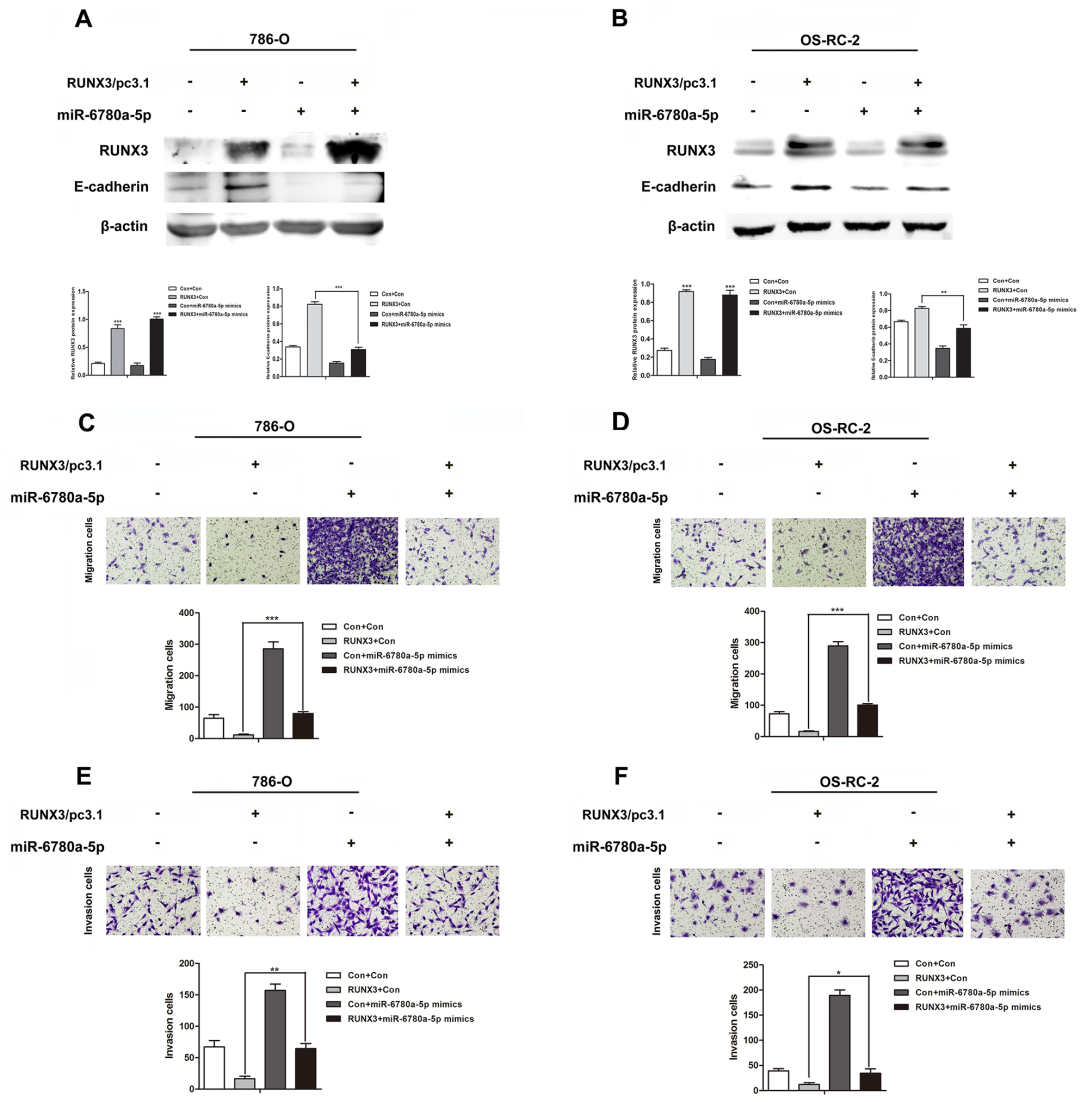
MiR-6780a-5p mimics abrogated RUNX3-mediated E-cadherin upregulation and RCC metastasis inhibition Western blot analysis showed that miR-6780a-5p mimics blocked RUNX3-mediated E-cadherin upregulation in 786-O and OS-RC-2 cells **A & B.** Cell migration **C & D.** and invasion **E & F.** assays in 786-O and OS-RC-2 cells infected by control, RUNX3/pc3.1, miR-6780a-5p mimics or RUNX3/pc3.1 with miR-6780a-5p mimics. Migration and invasion was greater in the RUNX3/pc3.1 plus miR-6780a-5p mimics group compared with the RUNX3/pc3.1 group Data are shown as means ±SD of three replicates (^*^P<0.05, ^**^P<0.01, ^***^P<0.001).

To determine whether RUNX3-mediated RCC metastasis inhibition was controlled by miR-6780a-5p induction, we upregulated miR-6780a-5p using mimics in RUNX3-overexpressing RCC cells. miR-6780a-5p upregulation attenuated RUNX3-induced E-cadherin expression (Figure 6A–6B) and abrogated RUNX3-mediated migration and invasion repression (Figure 6C–6F). These results suggested that the effects of RUNX3 on E-cadherin and cell metastasis were largely dependent on miR-6780a-5p inhibition.

## DISCUSSION

The effects of RUNX3 on tumorigenesis and metastasis have been widely studied in recent years [18]. RUNX3 has multiple functions and was demonstrated to play tumor suppressive roles in numerous human cancers, such as gastric cancer, RCC, colorectal cancer, breast cancer and melanoma [11, 18–20]. Hypermethylation of CpG island was demonstrated to be the key factor to lead to the downregulation of RUNX3 [21]. Additionally, point mutations *of RUNX3* were reported in certain cancers, including human gastric and bladder cancers [21, 22]. However, RUNX3 involvement in RCC progression and metastasis remains largely unknown. In the present study, we addressed whether RUNX3 promotes tumorigenesis and metastasis in renal cell carcinoma.

We used TMA technology and IHC to investigate the role of RUNX3 in RCC. RUNX3 was downregulated in RCC tumor tissue specimens as compared with normal renal tissues. We also analyzed relationships between RUNX3 expression and clinicopathological characteristics and found that downregulation of RUNX3 was related with depth of invasion (pT stage) and TNM stage. Analysis of Cox proportional hazards regression indicated that RUNX3 downregulation was correlated with poor patient OS and DSS. Thus, RUNX3 may play a significant role in RCC progression and may provide the opportunity to identify patients with RCC who are at high risk of unfavorable survival.

Decreased expression of certain tumor suppressive genes get the ability to promote cell motility and invasion [23], and we suspected that RUNX3 might inhibit renal cancer cell migration and invasion. Our results showed that downregulation of RUNX3 promoted cell migration and invasion through matrigel-coated chambers *in vitro*, whereas RUNX3 overexpression suppressed cell migration and invasion. As tumor cell migration and invasion are essential for metastasis, our findings hinted that RUNX3 may have the potential to regulate RCC metastasis. Previous research suggested that EMT is critical for the malignant transformation of tumors, especially metastasis [12, 14]. We examined E-cadherin and vimentin levels, the hallmarks of EMT, after RUNX3 overexpression or knockdown. While vimentin expression was not changed, RUNX3 positively regulated E-cadherin, which was consistent with RUNX3 activity *in vivo*. Ectopic expression of RUNX3 reportedly promoted E-cadherin expression, but had a negative effect on vimentin in hepatocellular carcinoma cells [24]. However, Liu, *et al.* indicated suppression of vimentin by RUNX3, but reported no change in E-cadherin expression in human gastric cancer cells [25]. Our results suggested that RUNX3 overexpression could not suppress vimentin, but did induce E-cadherin in human RCC. We speculate that there exist different EMT regulating mechanisms in different cancers.

E-cadherin expression is inversely correlated with several transcription factors, such as snail (SNAI1), slug (SNAI2) and twist homolog 1 (TWIST1) [14, 26, 27]. Unexpectedly, our data revealed that changes in RUNX3 level had no influence on SNAI2 or TWIST1 expression. We speculate that distinct expression profiles exist in SNAI2 or TWIST1. The specific type of cell or tissue involved may decide their contributions to EMT [28]. Thus, RUNX3 may regulate E-cadherin via different mechanisms in RCC, and further investigations are necessary to elucidate these mechanisms.

MicroRNAs are naturally occurring, small, non-coding, single-strand RNAs made up of 20-22 nucleotides (nt), and are gaining attention as potential regulators of EMT progression [29, 30]. miRNAs modulate gene expression posttranscriptionally by targeting the untranslated regions. This leads to degradation of the target mRNAs and/or translation inhibition [17]. Considerable research has been dedicated to determining whether specific miRNAs function as tumor suppressors or oncogenes [31, 32]. For instance, miR-9 upregulation, which can be seen in breast cancer, targets E-cadherin-encoding messenger RNA and induces a mesenchymal phenotype with increased migratory or invasive capacity [33, 34]. Yamasaki, *et al.* reported that miR-138 suppressed RCC cell metastasis by targeting vimentin [35]. Therefore we considered whether RUNX3 could regulate miRNA expression to downregulate E-cadherin and promote migration and invasion in RCC.

We used a miRNA array to perform miRNA analyses of 786-O RUNX3 knockdown and control cells, and detected 43 miRNAs upregulated more than two fold in RUNX3 knockdown cells. Only miR-6780a-5p was predicted to target E-cadherin. We found that RUNX3 bound to the miR-6780a-5p promoter directly and repressed expression of the miRNA. It is well established that RUNX3 could directly bind the promoter of miR-30a and promotes miR-30a expression [25].

To determine whether miR-6780a-5p regulates E-cadherin expression, we transfected renal cancer cells with miR-6780a-5p mimics. miR-6780a-5p overexpression decreased E-cadherin levels and promoted metastasis in human renal cancer cells. In contrast, miR-6780a-5p inhibition upregulated E-cadherin and suppressed metastasis. miR-6780a-5p could directly bind to the E-cadherin 3’UTR. To determine whether RUNX3 upregulated E-cadherin through miR-6780a-5p, we inserted miR-6780a-5p mimics into renal cancer cells stably overexpressing RUNX3. The miR-6780a-5p mimics abolished RUNX3-mediated E-cadherin upregulation and inhibition of renal cancer cell metastasis. These findings showed that RUNX3 upregulated E-cadherin and suppressed RCC metastasis by downregulating miR-6780a-5p expression.

In conclusion, we showed that RUNX3 downregulation is associated with RCC tumorigenesis and metastasis. Moreover, RUNX3 negatively regulates miR-6780a-5p expression by directly binding the miR-6780a-5p promoter, and miR-6780a-5p downregulates E-cadherin by directly binding its 3’UTR. RUNX3 may act as an effective prognostic indicator in RCC, and the pathways involved in RUNX3 activity may provide novel therapeutic targets for disease management.

## MATERIALS AND METHODS

### Patient samples

The study material includes 300 RCC and 35 adjacent normal renal tissues cases from the Affiliated Hospital of Xuzhou Medical College, treated between 2005 and 2008. All patients underwent surgery only or postoperative adjuvant therapy. The patients’ clinicopathologic information was obtained from the pathology department archive and was confirmed by hospital medical records. Follow-up information was obtained by reviewing patient medical records.

### Antibodies and reagents

Anti-RUNX3 mouse monoclonal antibodies were purchased from MBL (Medical and Biological Laboratories, Nagoya, Japan). Rabbit monoclonal antibodies to E-cadherin and vimentin were purchased from BD Biosciences (NJ, USA). Mouse anti-β-actin was from Boster Biotechnology (Wuhan, China). Rabbit antibodies to TWIST1 and SNAI2 were purchased from BIOSYNTHESIS (Beijing, China).

### Immunohistochemistry

IHC staining was performed as described previously [36]. The primary mouse anti-RUNX3 antibody (1:100), anti-E-cadherin and vimentin (1:100) were used. For TMA staining evaluation, immunoreactivity was assessed by two blinded independent observers using light microscopy (Olympus BX-51 light microscope), and images were collected by a Camedia Master C-3040 digital camera. RUNX3 expression was graded as positive when 10% of tumor cells showed immunopositivity. Biopsies with less than 10% tumor cells showing immunostaining were considered negative. The detailed conditions are described previously [37].

### Animals and cell lines

Female BALB/c nude mice, 6 weeks old, were purchased from the Shanghai Laboratory Animal Center (Shanghai, China) for studies approved by the Animal Care Committee of Xuzhou Medical College. Human RCC cell lines 786-O and OS-RC-2 were purchased from the Shanghai Institute of Biochemistry and Cell Biology, Chinese Academy of Sciences (Shanghai, China). 786-O and OS-RC-2 cells were cultured in RPMI1640 medium supplemented with 10% fetal calf serum (Invitrogen, Shanghai, China). Cells were in a 37°C humidified incubator with 95% air, 5% CO2.

### Transfection

pcDNA3.1 and RUNX3/pc3.1 expression plasmids were obtained from Dr Pei-Jung Lu (National Cheng-Kung University, Tainan, Taiwan). Transient transfection of plasmids into renal carcinoma cells was carried out using Lipofectamine 2000 transfection reagent (Invitrogen, Shanghai, China) following the manufacturer's protocol. Non-specific control siRNA or RUNX3 siRNA were purchased from Integrated Biotech Solutions (Shanghai, China). The sequences for the RUNX3 siRNA and control siRNA were 5’-CCUUCAAGGUGGUGGCAUUTT-3’ and 5’-UUCUCCGAACGUGUCACGUTT-3’, respectively. Cells were transfected with siRNA using siLentFect Lipid Reagent (Bio-Rad, Hercules, CA, USA) according to the manufacturer's instructions. Human miR-6780a-5p mimics, control mimics, inhibitors and control inhibitors were synthesized by Integrated Biotech Solutions (Shanghai, China).

For stable transfection, the lentiviral expression vectors LV5-Control and LV5-RUNX3 were obtained from Shanghai Gene Pharma Company (China). Lentiviruses were mixed with polybrene (5 mg/ml) and added 786-O cells. Positive clones were selected in puromycin (5 mg/ml). Stable RUNX3 transfectants were isolated after 2 weeks.

### Cell migration and invasion assays

Cell migration and invasion assays were performed using modified two chamber plates with a pore size of 8 μm. Transwell filter inserts with or without Matrigel (BD Biosciences) coating were used, respectively, for invasion and migration assays. Detailed conditions were described previously [38].

### Western blot analysis

Cell extracts were separated on a 10% SDS-polyacrylamide gel and proteins were transferred to nitrocellulose membranes and incubated overnight at 4°C with the following antibodies: mouse anti-RUNX3, β-actin, rabbit anti-E-cadherin, vimentin, SNAI2 or TWIST1. After incubation with peroxidase-coupled anti-mouse or anti-rabbit IgG at 37°C for 2 hours, membranes were washed and scanned using the Odyssey Two-Color Infrared Imaging System (LI-COR Biotechnology, Lincoln, Nebraska, USA). Each western blot was repeated three times.

### RNA extraction and real-time PCR

RNA extraction was carried out using Trizol (Invitrogen) according to the manufacturer's instructions. Real-time PCR amplification of RUNX3, E-cadherin and GAPDH was performed for 30 s at 95°C, followed by 40 cycles at 95°C for 5 s and annealing at 60°C for 34 s using an ABI PRISM 7500 Sequence Detection System (New York, USA). Target mRNA levels were calculated based on the CT method, normalized to GAPDH, and are expressed as a ratio of the percentage of gene copies to the GAPDH control. All reactions were run in triplicate. The primer sequences for each gene are as follows: RUNX3 Forward, 5’-TCTGTAAGGCCCAAAGTGGGTA-3’, Reverse, 5’-ACCTCAGCATGACAATATGTCACAA-3’; E-cadherin Forward, 5’-TTCCTCCCAATACATCT CCC-3’, Reverse, 5’-TTGATTTTGTAGTCACCCA CC-3’; GAPDH Forward, 5’-TGAAGGTCGGAGTCA ACGGATT-3’, Reverse, 5’-CCTGGAAGATGGTGATG GGATT-3’.

### Reporter vector construction and luciferase assay

The 204-bp miR-6780a-5p binding sequence at the human E-cadherin gene (CDH1) 3’ UTR was cloned into the XhoI and NotI restrictions sites downstream of the Renilla luciferase gene in the psiCHECK™-2 vector. Mutagenesis of the putative miR-6780a-5p binding sites was performed using the QuikChange site–directed mutagenesis kit. Primer sequences are listed in Figure 5. Cells were seeded in 48-well plates and transiently transfected with appropriate reporter plasmids and miRNA using Lipofectamine 2000. Cells were harvested and lysed after 48 h. Luciferase activity was measured using the Dual-Luciferase Reporter Assay System (Promega, Madison, WI, USA), normalized to renilla luciferase. For each plasmid construct, transfection experiments were performed in triplicate.

### Chromatin immunoprecipitation

786-O or OS-RC-2 cells transfected with control siRNA or RUNX3 siRNA were cross-linked by incubation in 1% (v/v) formaldehyde-containing medium for 10 min at 37°C, then sonicated to form soluble chromatin. A RUNX3 antibody was used to precipitate DNA fragments bound by their corresponding elements. The protein–DNA complex was collected using protein A Sepharose beads (Millipore) and then eluted and reverse crosslinked. After protease K treatment, samples were extracted with phenol/chloroform and precipitated with ethanol. Recovered DNA was re-suspended in TE buffer and amplified by PCR. Primers used for ChIP assay were as follows: forward 5’-CTGTTCCCTGTGCTCTGACC-3’ and reverse 5’-AGAGGGCTGATCTAAGGCAC-3’, and control primers for GAPDH.

### Mouse model

BALB/c nude mice were randomly divided into two groups of 8 mice each. LV5-Control and LV5-RUNX3 786-O cells were suspended in PBS. Mice were injected intravenously with 2.5×10^6^ 786-O cells in 0.2 ml of PBS via tail vein. After 2 months, mice were sacrificed and their lungs were resected and fixed in 10% buffered formalin. The number of metastatic nodules on the surface of each set of lungs was counted by visual inspection using a stereoscopic dissecting microscope.

### Statistical analysis

Data are expressed as means ± SD. Two-factor analysis of variance and the Dunnett's t-test were used to assess differences within treatment groups. For TMA, statistical analysis was performed with SPSS 20 software (SPSS, Inc, Chicago, IL). Associations between RUNX3 staining and RCC patient clinicopathologic parameters, including age, gender, tumor size, grade, pT status and TNM stage, were evaluated by two-sided Fisher's exact tests. The Kaplan-Meier method and log-rank test were used to evaluate the correlation between RUNX3 expression and patient survival. Hazard ratios (HR) and 95% confidence intervals (CI) were calculated using multivariate Cox proportional hazards regression models to analyze the independent impact of clinicopathologic factors and RUNX3 on survival. P<0.05 was considered statistically significant.
